# Supplementary material for: Genomic 3D compartments emerge from unfolding mitotic chromosomes
Source: Chromosoma. 2018 Oct 24;128(1):15–20. doi: 10.1007/s00412-018-0684-7 (PMC6394678; doi:10.1007/s00412-018-0684-7)
Supplement: Supplementary file 1 — (DOCX 1013 kb) [file 412_2018_684_MOESM1_ESM.docx]

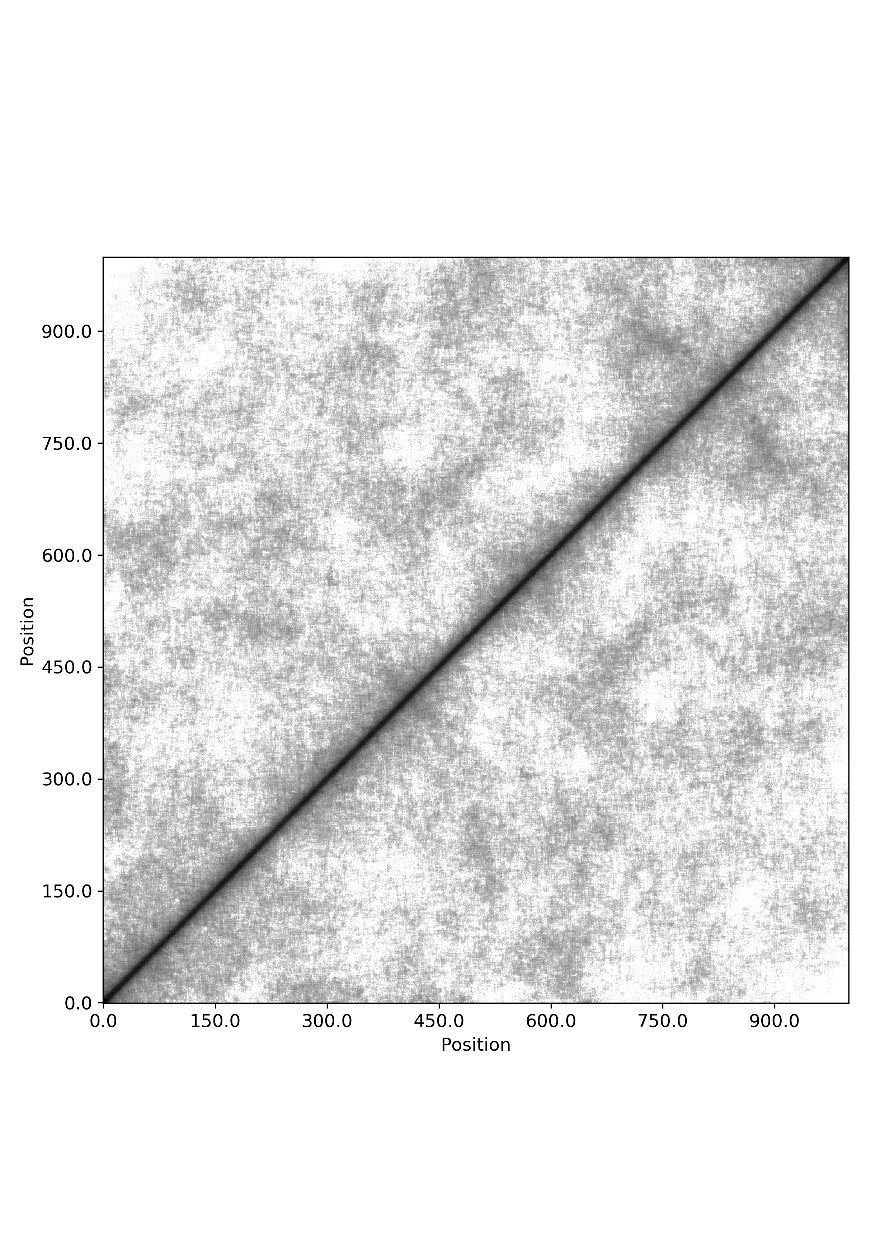


**Figure S1: Contact map of crumpled globules**. Average bead-bead contact map obtained from an ensemble of crumpled globules.


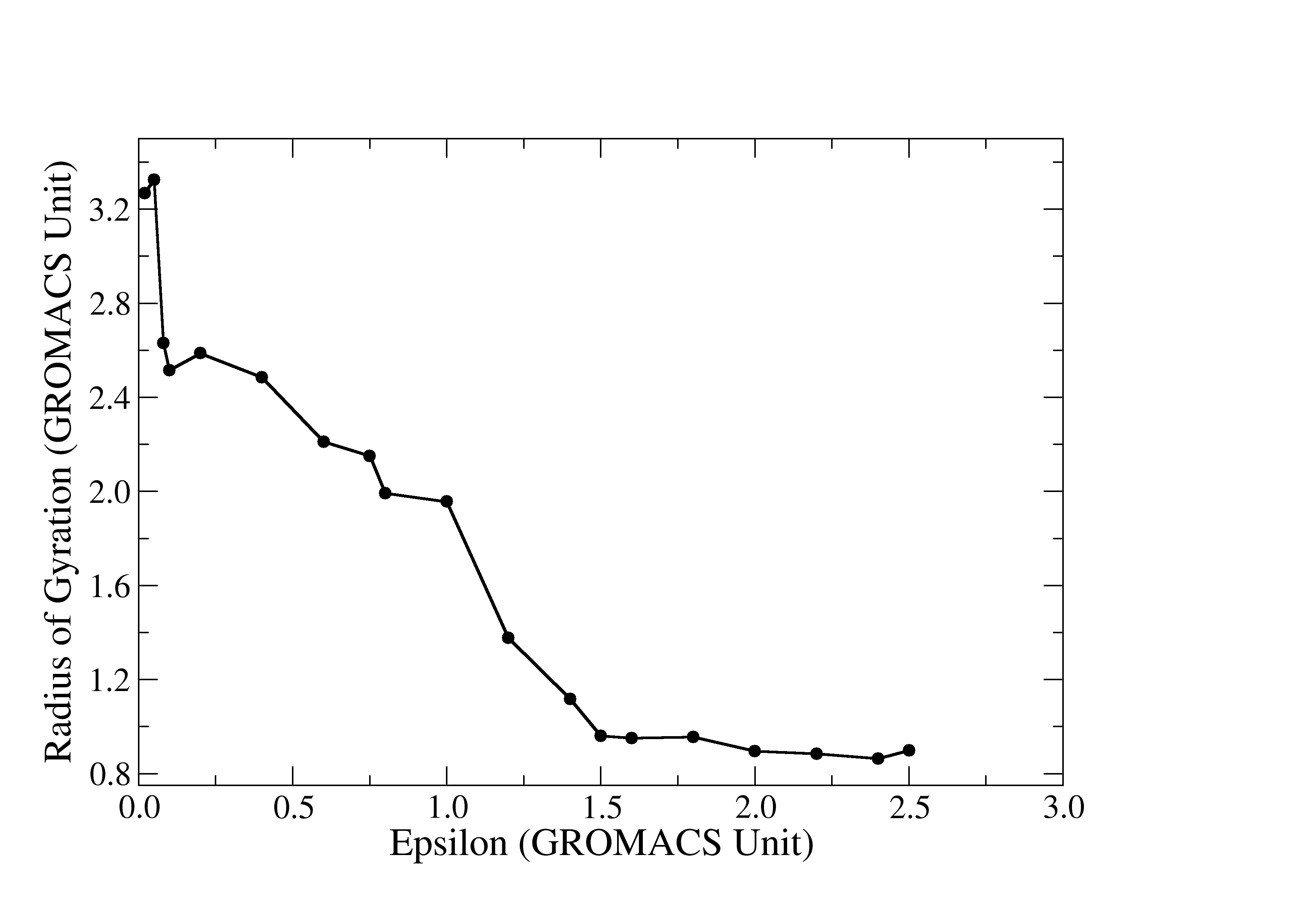


**Figure S2: The radius of gyration as a function of epsilon**. The black line is based on an average of 10 globule configurations at each epsilon value. In this simulation all beads in the polymer are identical.


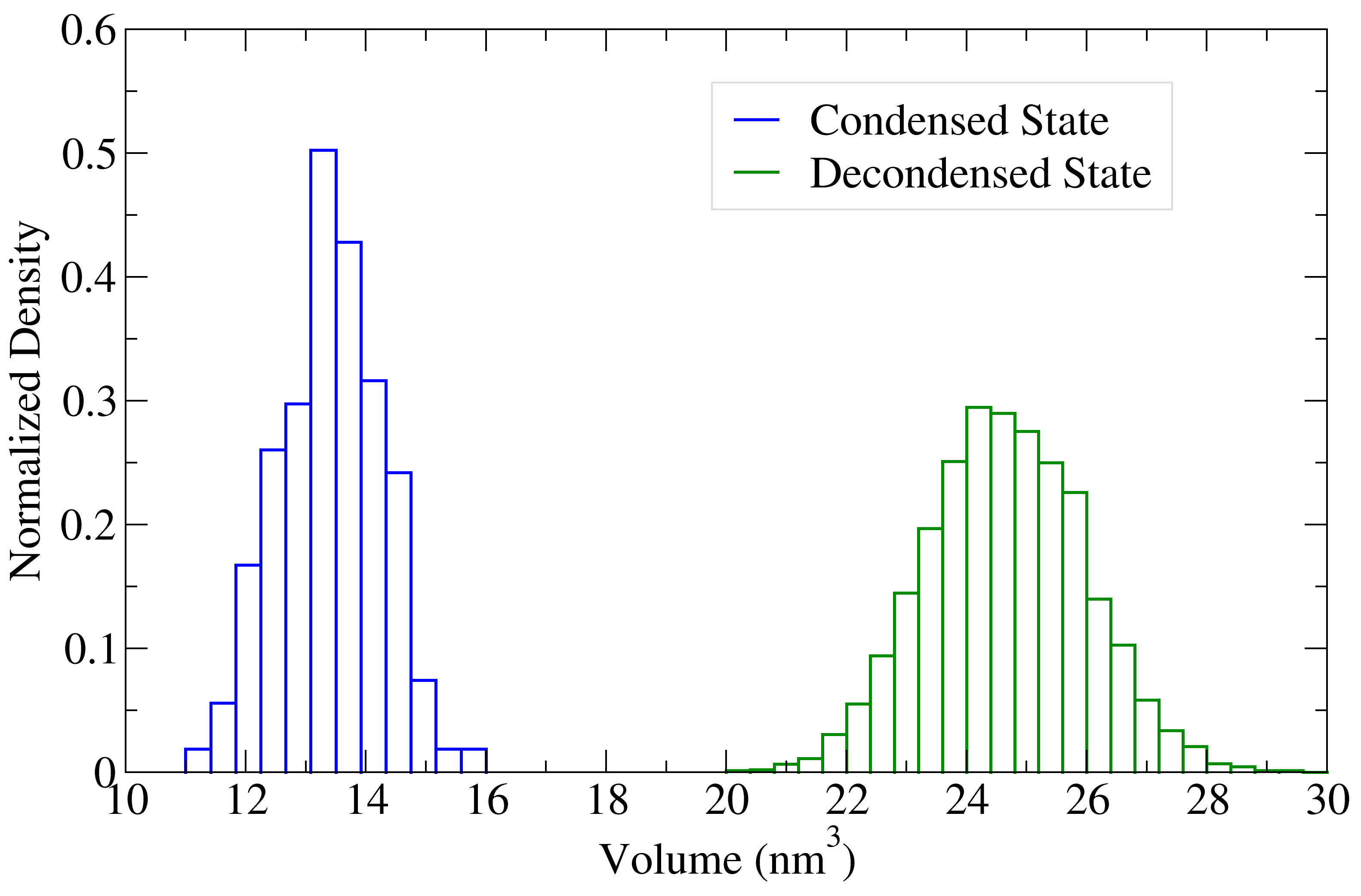


**Figure S3: Volume of the condensed and decondensed state**. Volume distribution for realized configurations of globules at condensed and decondensed states were calculated from the simulations. To calculate volume of the globules, we considered a probe of 2 Å to create a probe accessible surface enclosing the globule and calculated volume of this enclosed shape very similar to solvent accessible surface for macromolecules using the GROMACS tool g_sas. The probe of 2 Å radius was considered because 4 Å was used to calculate the contact map and with this radius, two beads in contact will be inside the same enclosed surface when rolling the probe over the globule.


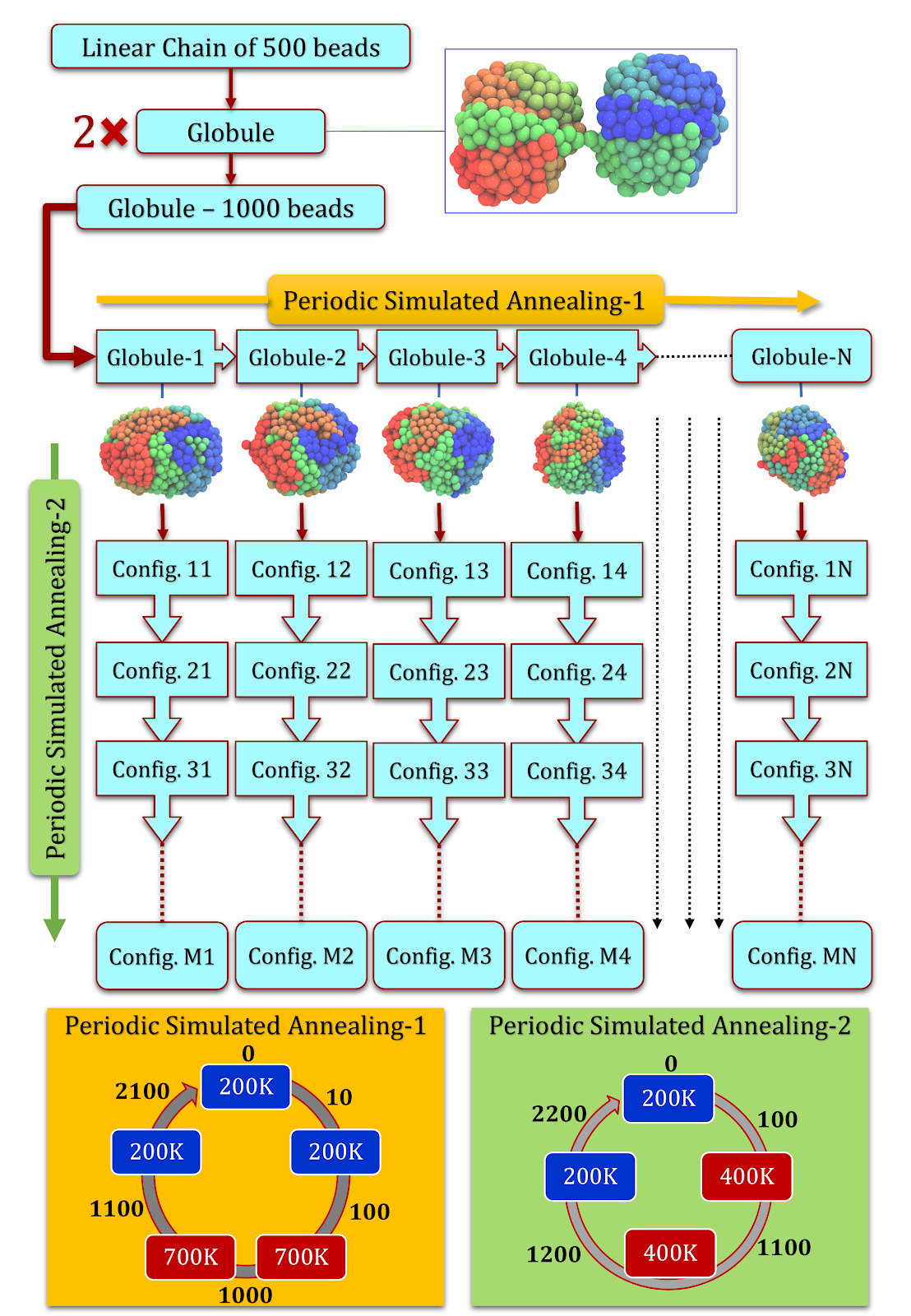


**Figure S4: A schematic illustration of the simulation procedure.** First we form two 500 bead globules, connect them, and merge them. The monomers are coloured from read through green to blue according to their linear order in the polymer. After merger, we used periodic simulated annealing (highlighted in orange) to generate diverse globule configurations. Subsequently, each globule was unfolded and periodic simulated annealing (green) was performed to generate diverse polymer configurations. At bottom we show the annealing process. The numbers inside the boxes show effective temperatures (Kelvin) while arrow indicates the number of MD steps.
